# Asymptomatic Bilateral Giant Multilocular Epididymal Cyst in a Middle-Aged Patient

**DOI:** 10.7759/cureus.24722

**Published:** 2022-05-04

**Authors:** Shubham Gupta, Sangita Shinde, Vishal Rathod, Raju K Shinde

**Affiliations:** 1 General Surgery, Datta Meghe Institute of Medical Sciences, Wardha, IND; 2 Pharmacology, Datta Meghe Institute of Medical Sciences, Wardha, IND; 3 Surgery, Datta Meghe Institute of Medical Sciences, Wardha, IND

**Keywords:** middle age, bilateral scrotal swelling, asymtomatic, multilocular, giant epididymal cyst

## Abstract

Bilateral multilocular huge epididymal cysts are a rare entity with few reports in the literature. Epididymal cysts are mostly found in middle-aged men with or without symptoms. We present the case of a 45-year-old man with asymptomatic bilateral scrotal swelling, which was clinically diagnosed as a right epididymal cyst with left hydrocele. However, an ultrasound of the scrotum revealed bilateral epididymal cysts with normal testes. Intraoperatively, it demonstrated bilateral huge epididymal cysts for which the patient underwent excision of bilateral epididymal cysts. Postoperatively, the patient is doing well on follow-up. Thus, it is concluded that when the epididymal cyst is larger than 10 mm or 1 cm and does not involute with time, surgery is indicated. In comparison, epididymal cysts smaller than 10 mm or 1 cm are managed conservatively.

## Introduction

Epididymal cyst or spermatocele is a fluid-filled sac arising from epididymis containing serous fluid [1]. It commonly affects middle-aged males [2]. They are mostly benign and unilateral in nature, although the bilateral presence is rare [3]. An asymptomatic, bilateral, and multilocular giant epididymal cyst is extremely rare, and only a few cases have been reported until today [4,5]. Approximately 30% of men have been diagnosed with small spermatoceles, while fewer have larger spermatoceles. The incidence of spermatocele increases with the age of the men [6]. Surgery is indicated if the cysts are larger than 10 mm or 1 cm. Otherwise, it is advised to manage conservatively [7]. Here, we present a rare case report of asymptomatic bilateral giant multilocular epididymal cysts in a middle-aged man.

## Case presentation

**Figure 1:**
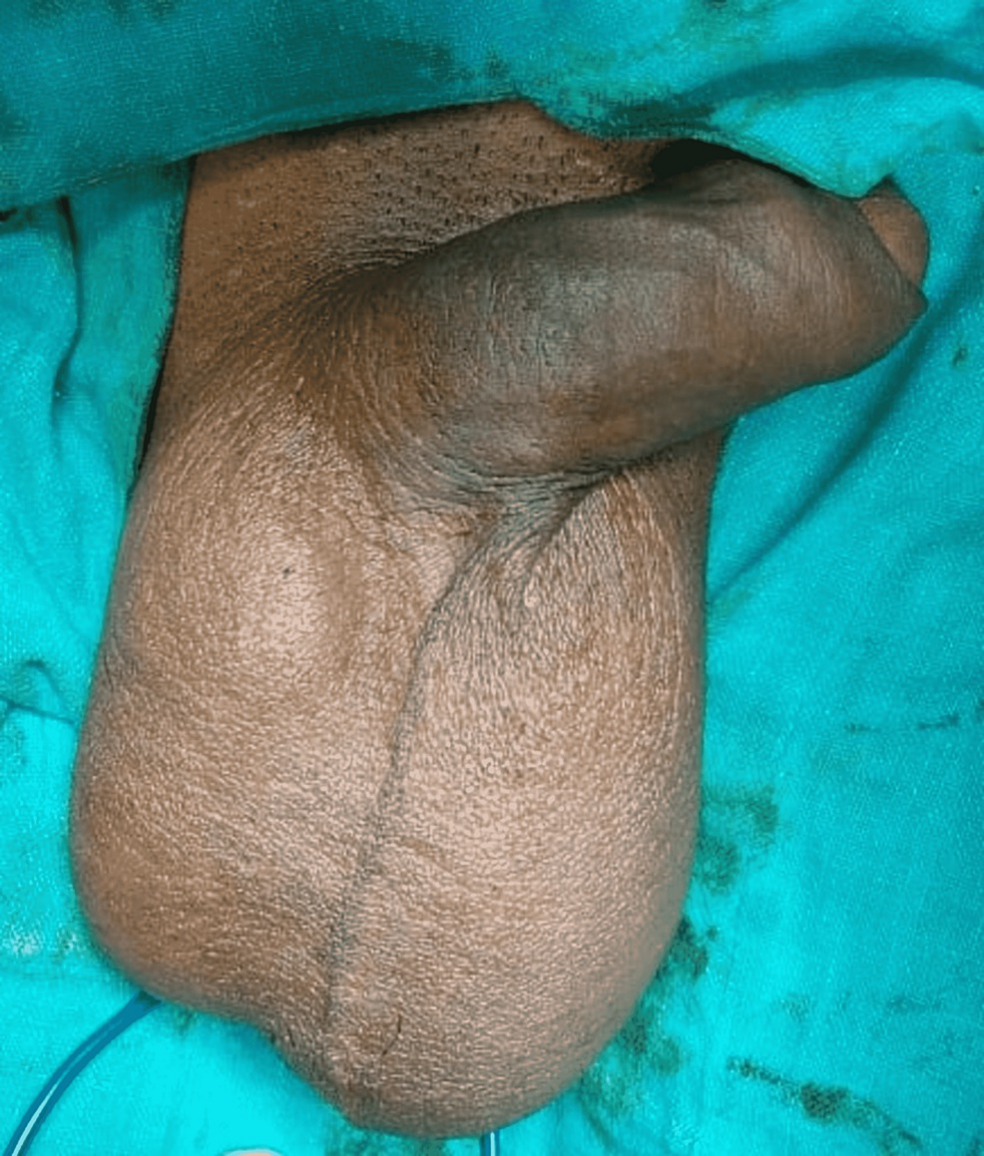
Clinical image of the patient with bilateral scrotal swellings.

A 45-year-old man presented to the surgical outpatient department (OPD) with complaints of bilateral scrotal swelling for eight months. The patient had no history of any trauma, pain, fever, urinary symptoms, or co-morbidities. Physical examination demonstrated non-reducible, non-tender, and bilateral scrotal swellings (left>right) (Figure 1) adjacent to both testes with fluctuation and transillumination tests positive. Both testes were palpable with normal size and location, and cough impulse was negative.

**Figure 2:**
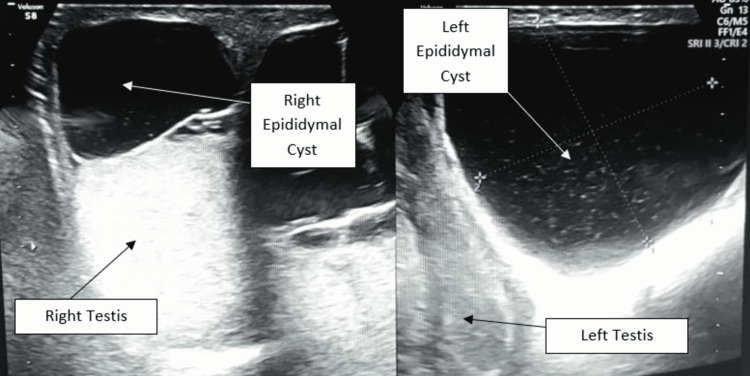
Scrotal ultrasonography images showing two large cystic swellings arising from both epididymal heads of size measuring 45 X 30 mm on the right and 100 X 70 mm on the left, with both testes normal in size, shape, and location.

Clinical differential diagnosis included left-sided secondary hydrocele with right-sided epididymal cysts and bilateral hydroceles of the spermatic cord. In addition, scrotal ultrasound showed two large cystic swellings arising from both epididymal heads of size measuring 45 X 30 mm on the right and 100 X 70 mm on the left (Figure 2).

**Figure 3:**
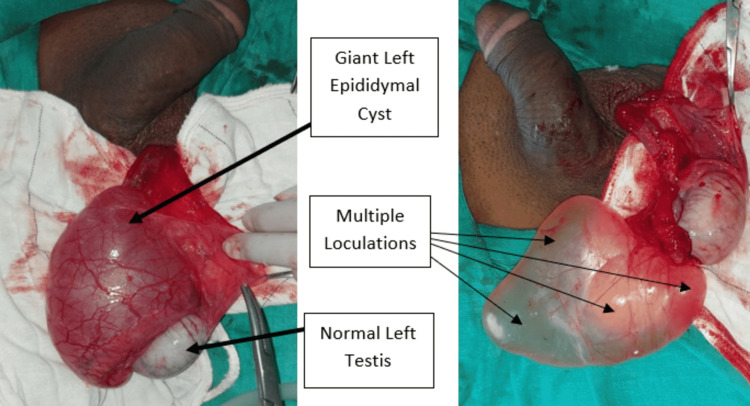
Intra-operative image of giant left epididymal cyst with multiple loculations and normal left testis.

**Figure 4:**
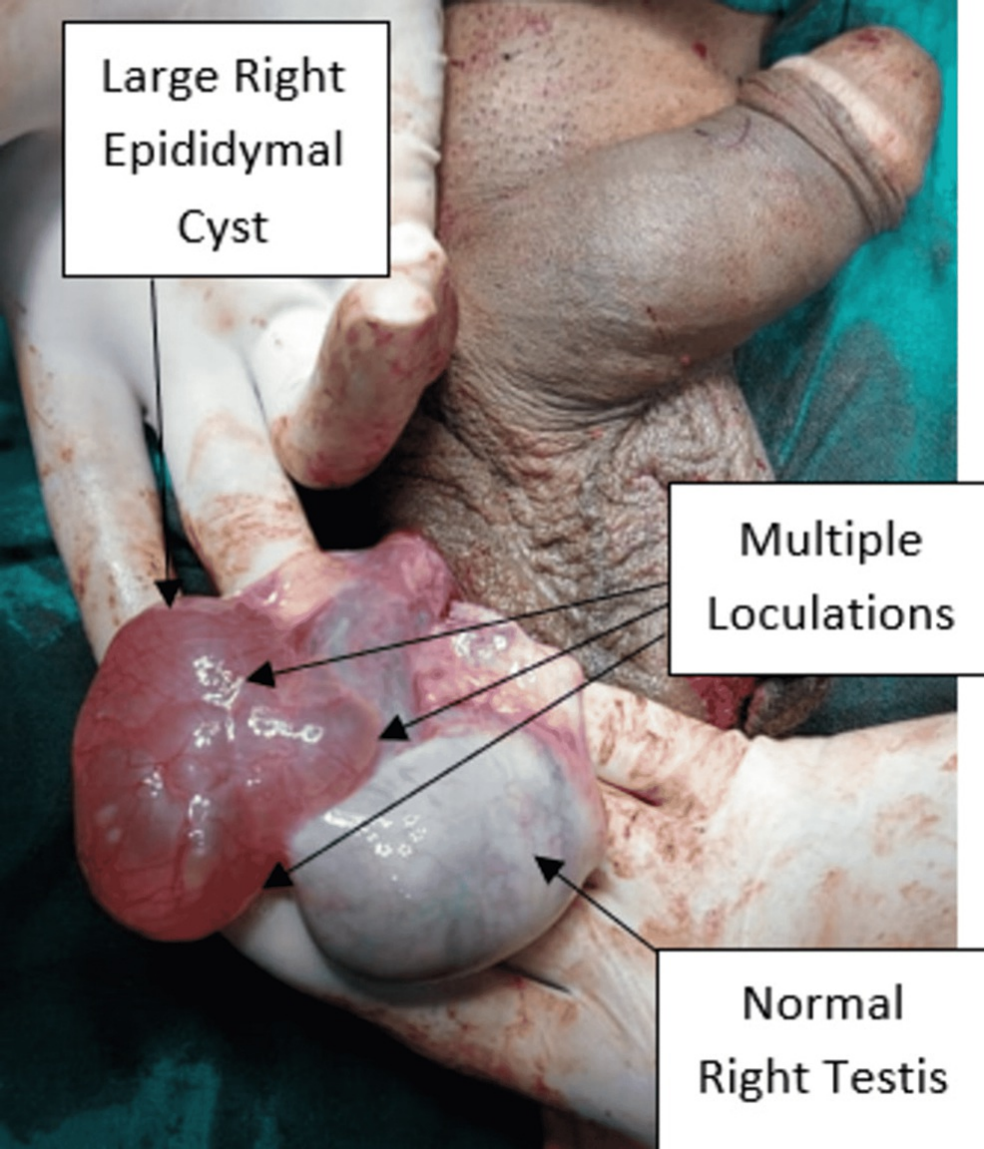
Intra-operative image of a large right epididymal cyst with multiple loculations and normal right testis.

**Figure 5:**
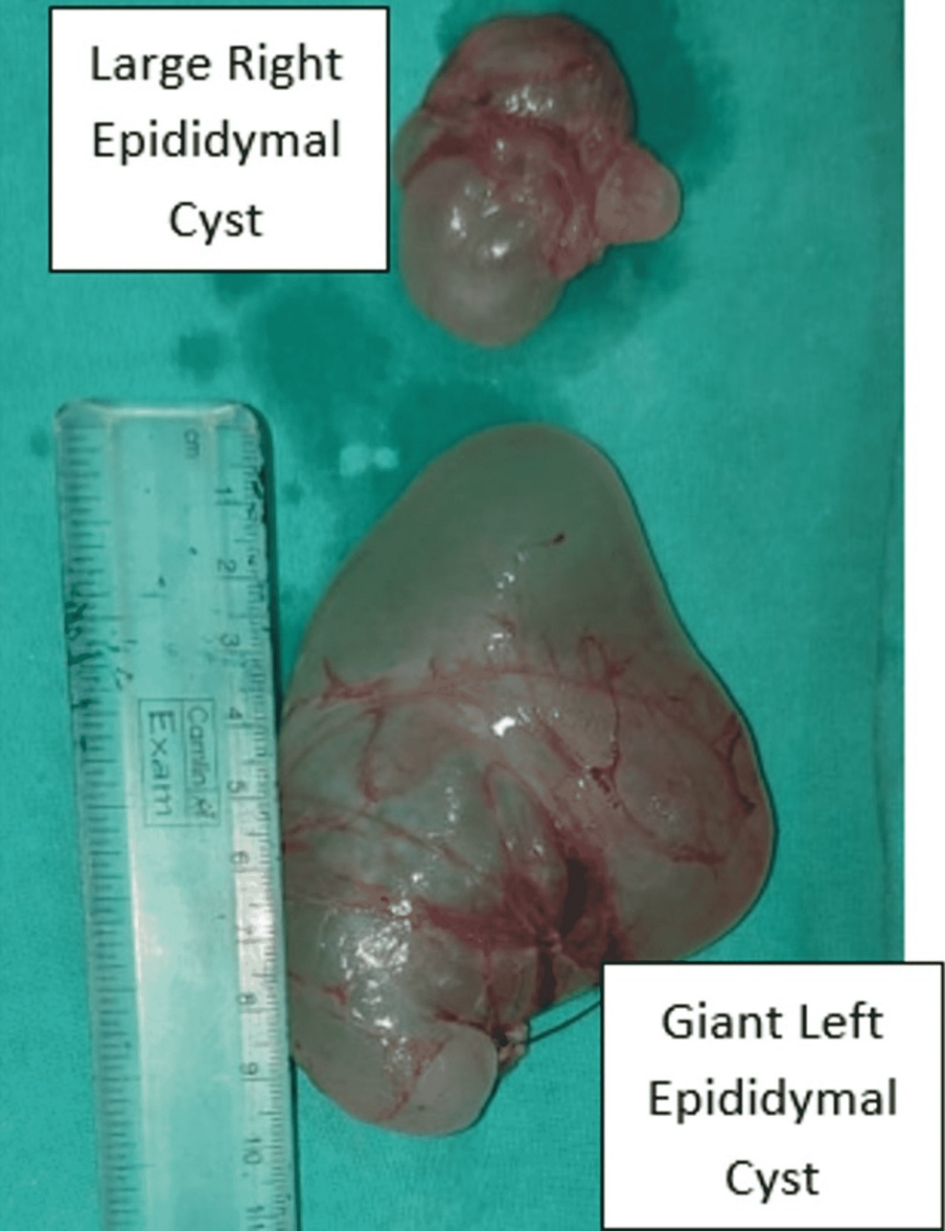
Postoperative images of specimen of both left (10 X 7 X 2.5 cm) and right (4.5 X 3 X 2 cm) epididymal cysts.

Both testes appeared normal in size, shape, and echogenicity with the normal color flow in the Doppler scan. Thus, the patient was taken for elective surgery. After tunica vaginalis exploration, a large thinned out translucent cyst was identified with multilocularity within, of size 10 X 7 X 2.5 cm on the left side (Figure 3) and 4.5 X 3 X 2 cm on the right side (Figures 4-5). The connection of the cysts was found to be attached to the heads of epididymis on both sides. The patient was discharged two days after the surgery and was advised to follow up for suture removal after seven days and maintain local hygiene over the suture site. 

## Discussion

An epididymal cyst is a fluid collection in either a single (unilocular) or more than one sac (multilocular) due to efferent epididymal tubules dilatation as a result of tubular obstruction [3,8]. They usually are unilateral and are found commonly amongst adult men. The incidence of either hydro or spermatocele diagnosed in specialized healthcare is approximately 100 per 100,000 men [9]. Bilateral epididymal cysts are significantly less frequent [4]. Multilocularity of epididymal cysts is rarer and rarely reported in the literature. Only Ushida H et al. [4] had reported multilocular bilateral synchronous epididymal cysts to date. Similar findings were noted in our case, too. A 45-year-old middle-aged gentleman had asymptomatic bilateral giant epididymal cysts with multilocularity arising from epididymal heads on both sides.

Furthermore, epididymal cysts can be symptomatic and can present with variable clinical presentations. A patient may narrate scrotal swellings as a third testicle. They may also present with testicular pain or orchialgia [10]. On clinical examination, the epididymal cyst is palpated as an extra testicular mass, usually smooth, round, and characteristically located adjacent to the testis [5]. These epididymal cysts are translucent in appearance since they contain clear fluid in them, but in some cases, it appears to be turbid due to the presence of spermatozoa within them [8]. In this case, clinically, the patient was thought to have a left secondary hydrocele with a right epididymal cyst preoperatively. In our patient, the size of the cyst was found to be larger as compared to the other similar reported cases [5,11-13] and was larger than the patient’s own testes as well. Additionally, our patient had no medical complaints or clinical symptoms related to the cysts preoperatively compared to the previously reported cases [11-17]. In this case, the patient did not have any symptoms like scrotal pain. It was only scrotal swelling that bothered the patient to undergo an ultrasound examination to confirm the diagnosis.

Ultrasound helps determine the location, site of origin, the content of the cyst, size, shape, and vascularity of the testes. The head of the epididymis is identified as the most common site of origin of the cyst, while the body and tail of the epididymis are rare sites [8,18]. Cystic tumors like epidermoid cyst (monodermal teratoma) of the testes and the adenomatoid tumor of epididymis could be considered a differential diagnosis [14].

Treatment options for epididymal cysts depend on the patient’s presentation, symptoms, and clinical findings. Most of the epididymal cysts involute with time [14]. Conservative management, sclerotherapy, aspiration of the cyst, and excision of the cyst could be various management or treatment options for epididymal cysts [8]. The surgical modality is used when the patient presents with acute symptoms like acute scrotal or inguinal pain, scrotal swelling, and redness. Primary excision is done for the removal of the cyst [19]. Surgery is indicated when the cyst is larger than 10 mm or 1 cm and does not involute with time [7]. Our patient had an asymptomatic giant bilateral multilocular epididymal cyst. He underwent excision of cysts bilaterally and was discharged after two days, and he was doing well on his follow-up.

## Conclusions

Conservative management still remains the initial choice of treatment for asymptomatic epididymal cyst with a size measuring < 1 cm, while surgical modality is the only mainstay of treatment for patients with acute symptomatology like intractable scrotal pain, swelling, and redness and also when the size of the cyst does not regress on its own instead increases with time.

From this rare case presentation, we would like to conclude that while dealing with the cases of small-to-medium scrotal swelling or small hydrocele, epididymal cysts should always be kept into consideration as a differential diagnosis.
